# Mesenchymal Cell Interaction with Ovarian Cancer Cells Triggers Pro-Metastatic Properties

**DOI:** 10.1371/journal.pone.0038340

**Published:** 2012-05-30

**Authors:** Raphael Lis, Cyril Touboul, Christophe M. Raynaud, Joel A. Malek, Karsten Suhre, Massoud Mirshahi, Arash Rafii

**Affiliations:** 1 Stem Cell and Microenvironment Laboratory, Weill Cornell Medical College in Qatar, Education City, Qatar Foundation, Doha, Qatar; 2 UMRS 872 INSERM, Université Pierre et Marie Curie, Equipe 18, Centre de Recherche des Cordeliers, Paris, France; 3 Genomic Core, Weill Cornell Medical College in Qatar, Education City, Qatar Foundation, Doha, Qatar; 4 Department of Physiology and Biophysics, Weill Cornell Medical College, New York, New York, United States of America; 5 Department Genetic Medicine, Weill Cornell Medical College, New York, New York, United States of America; Institut Gustave Roussy, France

## Abstract

Tumor microenvironement is an important actor of ovarian cancer progression but the relations between mesenchymal cells and ovarian cancer cells remain unclear. The objective of this study was to determine the ovarian cancer cells' biological modifications induced by mesenchymal cells. To address this issue, we used two different ovarian cancer cell lines (NIH:OVCAR3 and SKOV3) and co-cultured them with mesenchymal cells. Upon co-culture the different cell populations were sorted to study their transcriptome and biological properties. Transcriptomic analysis revealed three biological-function gene clusters were enriched upon contact with mesenchymal cells. These were related to the increase of metastatic abilities (adhesion, migration and invasion), proliferation and chemoresistance *in vitro*. Therefore, contact with the mesenchymal cell niche could increase metastatic initiation and expansion through modification of cancer cells. Taken together these findings suggest that pathways involved in hetero-cellular interaction may be targeted to disrupt the acquired pro-metastatic profile.

## Introduction

Most patients with ovarian cancer generally die from peritoneal disease [1]. Development of peritoneal carcinomatosis involves defined critical steps, including cell shedding, interaction and adhesion to mesothelial layer, and proliferation into the sub-mesothelium [2]. Several reports describe a pre-metastatic niche within the target organ, facilitating the initial survival of tumor cells [3], [4], [5].

Mesenchymal cells (MCs) are pluripotent cells that give rise to a variety of connective tissue cell types [6]. Bone Marrow-derived Mesenchymal Stem Cells (BM-MSC) are recruited in significant number to tumor sites where they contribute to invasion, metastasis, and resistance to chemotherapy of ovarian cancer cell lines [7], [8], [9], [10], [11]. MSC have been shown to contribute to ovarian cancer tumorigenicity through altered production of Bone Morphogenetic Protein (BMP2) leading to an increase of ovarian cancer cell (OCC) proliferation *in vitro* and *in vivo* [12]. While many studies focus on specific factors in the acquisition of metastatic profile, only few studies are assessing the global transcriptomic changes occurring in cancer cells upon their interaction with the mesenchymal stem cells (MSC) [13]. Zhang S et al.^13^ reported the modification of the prostate cancer cell transcriptome induced by interaction with MCs. The modifications defined a new pro-metastatic state. Understanding the pathways impacted by the interaction of cancer cells and their microenvironment and the induced global modifications is mandatory in order to be able to target microenvironment specific pathways [13].

Here, we investigate co-culture of two different types of OCC lines with MCs. We demonstrate that this interaction enhances metastatic abilities of OCC. Using transcriptomic analysis and functional assays; we demonstrate that genes related to cellular adherence, invasion, migration, proliferation and chemoresistance are modified upon OCC/MC contact in a cell line specific manner. Our results suggest that specific pathways may be targeted to disrupt the acquired pro-metastatic profile.

## Materials and Methods

### Cell Culture

Ovarian cancer cell lines SKOV3 (HTB-77) and OVCAR3 (HTB-161) were purchased from ATCC and maintained in culture following ATCC recommendations (DMEM high glucose [Hyclone, Thermo Scientific], 10% FBS [Hyclone, Thermo Scientific], 1% Penicillin-Streptomycin-Amphotericyn B solution [Sigma], 1X Non Essential Amino-Acid [Hyclone, Thermo Scientific]). The ovarian cancer cell lines were stably transduced by lentiviral vectors encoding eGFP (Genethon, Evry). Mesenchymal cells were purchased from Stem Cells, Inc (MSC-001F, Vancouver, CA) maintained and expanded in culture using MesenCult® MSC Basal Medium completed with Mesenchymal Stem Cell Stimulatory Supplements (Stem cell Inc, Vancouver, CA). Their ability to differentiate in adipocytes, osteoblasts and chnodrocytes was verified as per the supplier instructions (data not shown).

### Co-Cultures

We established co-cultures of eGFP-OCC with BM-MC at ratio of 1∶2 for 24 hours. OCC were differentiated from BM-MC based on their eGFP and Ep-Cam. The different cell populations were sorted using Fluorescence Activated Cell Sorting (FACS).

### Fluorescent activated cell sorting

Cells were harvested and blocked in PBS-5%FBS-1%BSA-10%FcR Blocking Reagent (Myltenyi Biotec). Single-cell suspension was analyzed and sorted on SORP FACSAria2 (BD Biosciences). Data were processed with FACSDiva 6.3 (BD Biosciences). Doublets were excluded by FSC-W × FSC-H and SSC-W × SSC-H analysis, single stained channels were used for compensation, and fluorophore minus one (FMO) controls were used for gating. eGFP fluorescence was acquired with 488 nm blue laser and 510/50 nm emission, 50 000 events were acquired per sample. Charts display the median of fluorescence intensity (mfi) relative to control. During cell-sorting purity-phase mask was applied. OCC monocultures were processed and sorted as controls.

### Gene expression analysis

Upon cell sorting mRNA was isolated using Trizol reagent followed by purification using RNAeasy extraction kit from Qiagen. 200 ng of total RNA were analyzed on Affymetrix GeneChip Human Genome U133 Plus 2.0 Array. Data were analyzed using Partek software (St Louis, MO). Class comparison between different conditions (three biological replicates) was performed to identify gene expression changes with significant expression differences and two-fold increased or decreased expression [14], [15], [16]. Principal component analysis (PCA) were performed using Partek with the standard settings. Statistical comparisons for microarray data were calculated using two-tailed Students t-test. Benjamini-Hochberg correction was applied to limit positive false discovery rate to 5%. Statistical comparisons for categorical data were achieved using Chi-squared test. Correlations were performed using Pearson correlation. All other statistical comparisons were calculated using two-tailed t-test.

### Ingenuity Pathway Analysis

We used Ingenuity Pathway Analysis software (IPA) (Ingenuity Systems, Redwood City, CA) for network analysis of genes that were differentially regulated upon co-culture. We defined global gene lists representing IPA keywords: “Metastasis”, “Proliferation of cell lines” and “Cell death of tumor cell line”. All edges are supported by at least one reference from the literature, textbook, or canonical information stored in the Ingenuity Pathways knowledge database. *P-*values for enrichment of biological functions were generated based on the hypergeometric distribution and calculated with the right-tailed Fisher's exact for 2×2 contingency tables as implemented in Ingenuity.

### Adherence assay

96 well-plates were coated with Matrigel (BD-Biosciences). 20 000 OCC-eGFP were seeded per well and incubated in 5%CO2 incubator at 37°C for 15, 30 and 60 minutes. Non-adherent cells were washed away. Adherent cells were quantified using fluorescence plate reader (Wallac, Perkin Elmer) (488 nm excitation, 510 emission). All functional experiments were performed in biological triplicates.

### Migration/Invasion assays

8 μm pores transwell permeable supports were coated with Matrigel. 50 000 viable OCC (sorted from co- and monoculture) were seeded per well and incubated in 5%CO2 at 37°C for 12 and 24 hours. OCC migration and invasion was then assessed using a fluorescence plate reader (Wallac, Perkin Elmer).

### Proliferation assay

5000 OCC were seeded in monoculture or on MSC-mOrange monolayer in a serum free/cytokine free media. OCC were counted every two days during 14 were coated with Matrigel. 50 000 days under a microscope using GFP fluorescence. Each day ten fields were quantified.

### Chemoresistance

100000 OCC were seeded in monoculture or with BM-MSC in 1∶2 ratio. Mono- and co-culture were treated with 90 µM Cisplatinum and 6 µM Paclitaxel (Sigma) for 24 h. Cells were then stained with Calcein Red-Orange, LIVE/DEAD Aqua blue (Invitrogen, Molecular Probes), CD73-APC (Biolegend, clone: AD2) according to manufacturer's instructions and analyzed by FACS. OCC were defined as cells GFP+CD73-, living OCC were defined as calcein Red-Orange+ LIVE/DEAD aqua-. 50 000 events were acquired per sample.

### Statistical analysis

Student-t tests, Fisher exact tests and chi-square tests were performed as appropriate. All p-values are two-sided with statistical significance evaluated at the 0.05 alpha levels. Ninety-five percent confidence intervals (95% CI) were calculated to assess the precision of the obtained estimates. All statistical analysis was done using the data analysis plug-in of Microsoft Excel 2008.

## Results

### Modification of the OCC transcriptome upon interaction with MC

**Figure 1:**
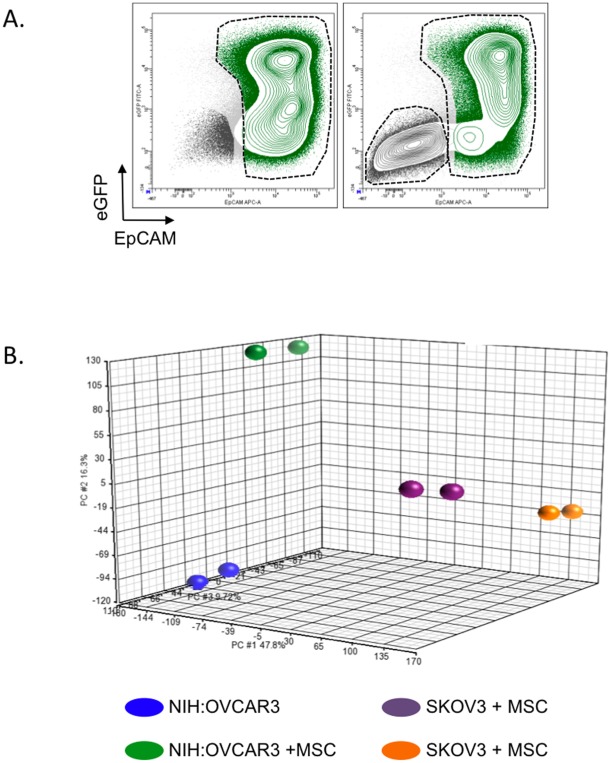
Modification of OCC transcriptome upon interaction with MC. A. Contour plot showing a typical discrimination of OCC and MC by FACS. OCC were defined as eGFP+EpCAM+ (green population) whereas MCs were defined as eGFP-EpCAM- (dark grey population). **B.** PCA analysis for the ovarian cancer cells lines alone or post-contact with the Mesenchymal cells.

In order to investigate modifications of OCC transcriptome upon MC contact, we designed a coculture model in which OCC are cultivated alone (control condition) or in presence of MC (test condition) during 24 h in a serum free, cytokine free medium. OCC (control and test conditions) were then sorted by FACS based on their co-expression of the epithelial cell adhesion molecule (EpCAM, CD326) and the enhanced-Green Fluorescent Protein (eGFP) (Figure 1.A).

Upon MC contact, the transcriptome of the two cell lines we tested (NIH:OVCAR3-eGFP and SKOV3-eGFP) was dramatically modified (Figure 1.B). NIH:OVCAR3 significantly upregulated 26 genes and downregulated 10 genes (Table S1); SKOV3 significantly upregulated 18 genes and downregulated 3 genes (Table S2). Changes observed in the two ovarian cancer cell lines did not allow us to establish a common gene signature upon MC contact (Figure 1.B, Table S1, Table S2). However using Ingenuity Pathway Analysis software, we were able to identify three biological function clusters enriched upon contact with MC: “Metastasis” (NIH:OVCAR3, p = 6.48*10^−6^; SKOV3, p = 5.42*10^−5^), “Proliferation of cell lines” (NIH:OVCAR3, p = 4.36.48*10^−7^; SKOV3, p = 6.79*10^−6^) and “Cell death of tumor cell line” (NIH:OVCAR3, p = 5.68*10^−8^; SKOV3, p = 7.62*10^−5^). These clusters were shared between NIH:OVCAR3-eGFP and SKOV3-eGFP (Table 1).

**Table 1:** MC contact enriches biological function gene clusters.

	OVCAR3-eGFP				SKOV3-eGFP			
Biological function	ID	Gene Name	Gene Symbol	Fold Change relative to the control	ID	Gene Name	Gene Symbol	Fold Change relative to the control
Metastasis	221577_x_at	Growth Diffentiation Factor 15	GDF15	**27.920**	206336_at	Chemokine (C-X-C motif) ligand 6	CXCL6	**15.511**
	217678_at	Solute carrier family 7 anionic amino acid transporter light chain, xc-system), member 11	SLC7A11	**15.653**	201438_at	Collagen alpha-3(VI) chain	COL6A3	**10.121**
	202672_s_at	Activating transcription factor 3	ATF3	**12.425**	212942_s_at	KIAA1199	KIAA1199	**9.958**
	201010_s_at	Thioredoxin interacting protein	TXNIP	**9.983**	216598_s_at	Chemokine (C-C motif) ligand 2	CCL2	**8.275**
	212314_at	sel-1 suppressor of lin-12-like 3	SEL1L3	**5.560**	228128_x_at	Pregnancy-associated plasma protein A	PAPPA	**7.330**
	200953_s_at	cyclin D2	CCND2	**5.423**	205828_at	Matrix metalloproteinase-3	MMP3	**5.878**
	210105_s_at	FYN oncogene related to SRC, FGR, YES	FYN	**5.237**				
	201438_at	Collagen alpha-3(VI) chain	COL6A3	**−9.575**				
	211719_x_at	Fibronectin 1	FN1	**−10.982**				
Proliferation of cell lines	221577_x_at	Growth Diffentiation Factor 15	GDF15	**27.920**	1556499_s_at	Collagen, type I, alpha 1	COL1A1	**31.150**
	217678_at	Solute carrier family 7 anionic amino acid transporter light chain, xc-system), member 11	SLC7A11	**15.653**	202291_s_at	Matrix gla protein	MGP	**16.068**
	206085_s_at		CTH	**14.916**	216598_s_at	Chemokine (C-C motif) ligand 2	CCL2	**8.275**
	202672_s_at	Activating transcription factor 3	ATF3	**12.425**	204051_s_at	Secreted frizzled-related protein 4	SFRP4	**7.872**
	201010_s_at	thioredoxin interacting protein	TXNIP	**9.983**	228128_x_at	Pregnancy-associated plasma protein A	PAPPA	**7.330**
	208763_s_at	TSC22 domain family, member 3	TSC22D3	**9.287**	228335_at	Claudin-11	CLDN11	**6.752**
	203725_at	growth arrest and DNA-damage-inducible, alpha	GADD45A	**8.742**	212667_at	Secreted Protein Acidic and Rich in Cysteine	SPARC	**6.460**
	203543_s_at	Kruppel-like factor 9	KLF9	**7.605**	205828_at	Matrix metalloproteinase-3	MMP3	**5.878**
	203140_at	B-cell CLL/lymphoma 6	BCL6	**5.544**	209821_at	Interleukin 33	IL33	**5.069**
	200953_s_at	cyclin D2	CCND2	**5.423**				
	212501_at	CCAAT/enhancer binding protein (C/EBP), beta	CEBPB (includes EG:1051)	**5.386**				
	210105_s_at	FYN oncogene related to SRC, FGR, YES	FYN	**5.237**				
	202581_at	heat shock 70kDa protein 1A/1B	HSPA1A/HSPA1B	**−5.105**				
	1556499_s_at	Collagen, type I, alpha 1	COL1A1	**−9.175**				
	211719_x_at	Fibronectin 1	FN1	**−10.982**				
	200665_s_at	Secreted Protein Acidic and Rich in Cysteine	SPARC	**−11.110**				
Cell Death of tumor cell line	221577_x_at	Growth Diffentiation Factor 15	GDF15	**27.920**	216598_s_at	Chemokine (C-C motif) ligand 2	CCL2	**8.275**
	205047_s_at	asparagie synthetase (glutamine-hydrolyzing)	ASNS	**23.756**	204051_s_at	Secreted frizzled-related protein 4	SFRP4	**7.872**
	217678_at	Solute carrier family 7 anionic amino acid transporter light chain, xc-system), member 11	SLC7A11	**15.653**	212667_at	Secreted Protein Acidic and Rich in Cysteine	SPARC	**6.460**
	202672_s_at	Activating transcription factor 3	ATF3	**12.425**	208998_at	Uncoupling Protein 2	UCP2	**−5.162**
	209383_at	DNA-damage-inducible transcript 3	DDIT3	**9.775**				
	203725_at	growth arrest and DNA-damage-inducible, alpha		**8.742**				
	202887_s_at	DNA-damage-inducible transcript 4	DDIT4	**6.046**				
	203140_at	B-cell CLL/lymphoma 6	BCL6	**5.544**				
	212501_at	CCAAT/enhancer binding protein (C/EBP), beta	CEBPB (includes EG:1051)	**5.386**				
	202581_at	heat shock 70kDa protein 1A/1B	HSPA1A/HSPA1B	**−5.105**				
	210338_s_at	heat shock 70kDa protein 8	HSPA8	**−7.289**				
	211719_x_at	Fibronectin 1	FN1	**−10.982**				
	200665_s_at	Secreted Protein Acidic and Rich in Cysteine	SPARC	**−11.110**				

Table showing the biological clusters identified by IPA analysis. p-values for the different clusters are: “Metastasis” (NIH:OVCAR3, p = 6.48*10^−6^; SKOV3, p = 5.42*10^−5^), “Proliferation of cell lines” (NIH:OVCAR3, p = 4.36.48*10^−7^; SKOV3, p = 6.79*10^−6^) and “Cell death of tumor cell line” (NIH:OVCAR3, p = 5.68*10^−8^; SKOV3, p = 7.62*10^−5^). These clusters were shared between NIH:OVCAR3-eGFP and SKOV3-eGFP.

Taken together these data indicate that despite the lack of overlapping regarding the genes up-or down-regulated by MC contact, the same biological function clusters were enriched in the two ovarian cancer cell line we tested. In this article, we set forth the idea that MC exert a pro-metastatic effect on OCC, therefore we decided to validate the biological function clusters described in Table 1 with *in vitro* experimentations.

### MCs increase OCC metastasis biological function: adherence, migration and invasion

**Figure 2:**
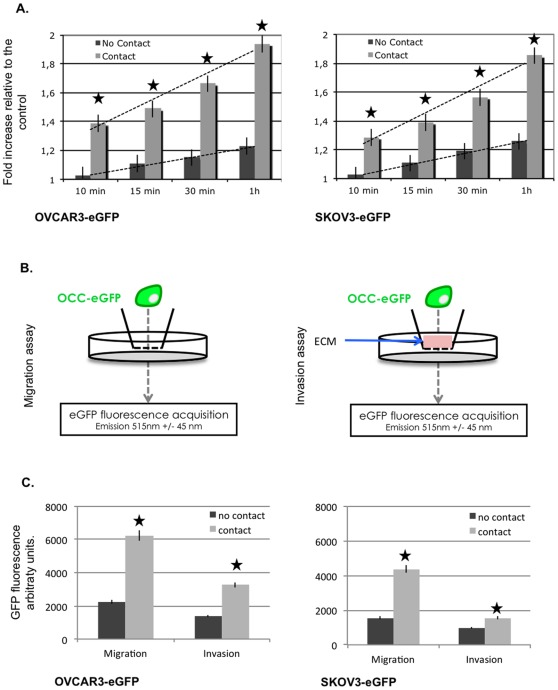
MCs increase OCC metastasis biological function: adherence, migration and invasion. **A.** OCC-eGFP (NIH:OVCAR3, left chart and SKOV3, right chart) were seeded on a Matrigel (BD Biosciences)-coated well for 10 min, 15 min, 30 min and 1 hour. Increased adherence to the ECM is observed when OCC were preemptively cultivated with MC (light grey bars) compared to the control (dark grey bars). **B.** Scheme representing the invasion and migration assay. **C.** Sorted OCC were seeded on (un) coated transwells and GFP signal of each well under the coated membrane was acquired after 24 h. Increased migration and invasion through the ECM is observed when OCC were preemptively cultivated with MC (light grey bars) compared to the control (dark grey bars). SEM are represented, n = 3, | p<0.05 t-Student Test.

Peritoneal metastasis initiation involves adherence, migration, and invasion through the mesothelium. Using IPA software, we identified a biological function cluster: “metastasis”, which was enriched upon ovarian cancer cell contact with MC. We therefore investigated the biological effect corresponding to this genomic pro-metastatic modification performing *in vitro* migration, adherence and invasion assays. Upon 24 hours of co-culture with MC, OCC (EpCAM+ eGFP+) were sorted (Figure 1A). We then tested OCC ability' to adhere to ECM. OCC (NIH:OVCAR3 and SKOV3) were seeded on a Matrigel (BD Biosciences)-coated well for 10 min, 15 min, 30 min and 1 hour. We defined the adherence to the ECM as the residual GFP fluorescence we were able to acquire following PBS washing. As displayed in Figure 2.A, both ovarian cancer cell line show an increased adherence to the ECM: 1.38 fold increase relative to the control for NIH:OVCAR3-eGFP at 10 min, and 1.94 fold increase at 1 h, 1.28 and 1.85 fold for SKOV3-eGFP at 10 min and 1 hour respectively.

To perform migration assay, we seeded 8 µm transwell with the OCC sorted upon 24 h of co-culture with MC. We tested their ability to migrate through the transwell insert and measured the GFP signal of each well after 24 h. We observed a 2 fold and 2.5 fold increased migration with NIH:OVCAR3 and SKOV3 after MC contact, respectively.

To perform invasion assay, we used Matrigel (BD Biosciences)-coated 8 µm transwells. Sorted OCC were seeded on coated transwells and GFP signal of each well under the coated membrane was acquired after 24 h. Cell migration was increased by 2.5 times and 1.5 times for NIH:OVCAR3 and SKOV3 after MC contact, respectively.

We demonstrated here, that MC through direct interaction with OCC were able to change drastically OCC behavior. Following MC contact OCC displayed a greater adherence to the ECM, a faster migration and a more efficient invasion through the ECM. Taken all together these observations emphasize the role of MC, to enhance at both the transcriptional and functional level the metastatic potential of OCC.

### Mesenchymal cells sustain ovarian cancer cells proliferation

**Figure 3:**
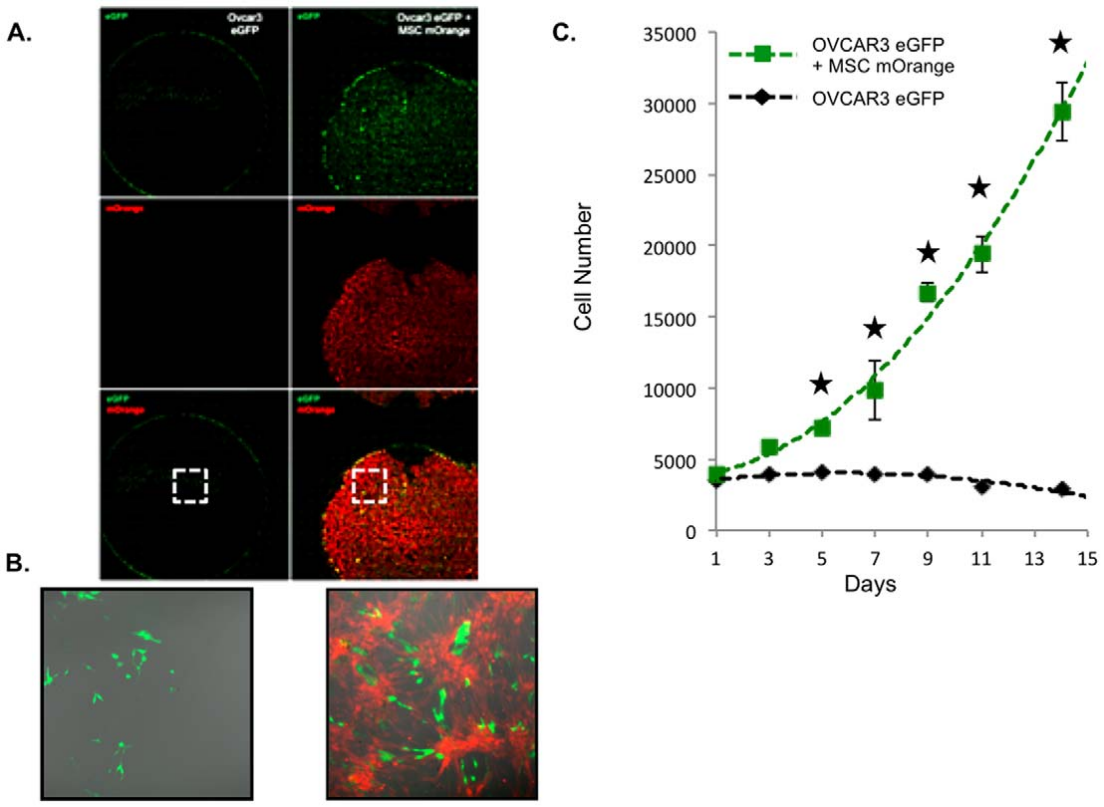
MC sustains OCC proliferation in a serum free cytokine free context. A. OCC were cultivated on a MC expressing mOrange or direclty on the plastic dish. Proliferation assay was carried out in a serum free-cytokine free media. Pictures were taken every two days, and GFP cells were quantified. **B.** Detail of the well on day 14, we can notice OCC display a normal morphology. **C.** Chart displaying cell count carried over 14 days. OVCAR3 eGFP when seeded on a MC are able to sustain cell cycle in a serum free cytokine free media. Similar results were obtained with SKOV3 (data not shown). SEM are represented, n = 3, | p<0.05 t-Student Test.

Following mesothelial infiltration, development of peritoneal carcinosis involves proliferation of ovarian cancer cells within their surrounding stroma. We clustered genes based on their biological function, and demonstrated that genes involved in proliferation of cell lines were enriched upon MC contact with OCC (Table 1). We therefore investigated the ability of the MC to sustain OCC proliferation. Since the use of serum in order could greatly hamper the study of the effect of the microenvironment on the ovarian cancer cell, we performed the proliferation assay in a in a serum free, cytokine free context. OCC-eGFP (NIH:OVCAR3 and SKOV3) were cultured alone or on a MC-mOrange monolayer. We observed that MC sustained cancer cell growth at least for 15 days while OCC were quiescent in their absence (Figure 3A–C).

### MC induce Chemoresistance of ovarian cancer cells

The treatment of ovarian cancer with peritoneal carcinosis, i.e. advanced stage disease, includes chemotherapy before or after surgery. We clustered genes based on their biological function, and demonstrated that genes involved in “Cell death of tumor cell line” of cell lines were enriched upon MC contact with OCC (Table 1). We therefore investigated whether MC are capable of promoting OCC resistance to chemotherapy.

**Figure 4:**
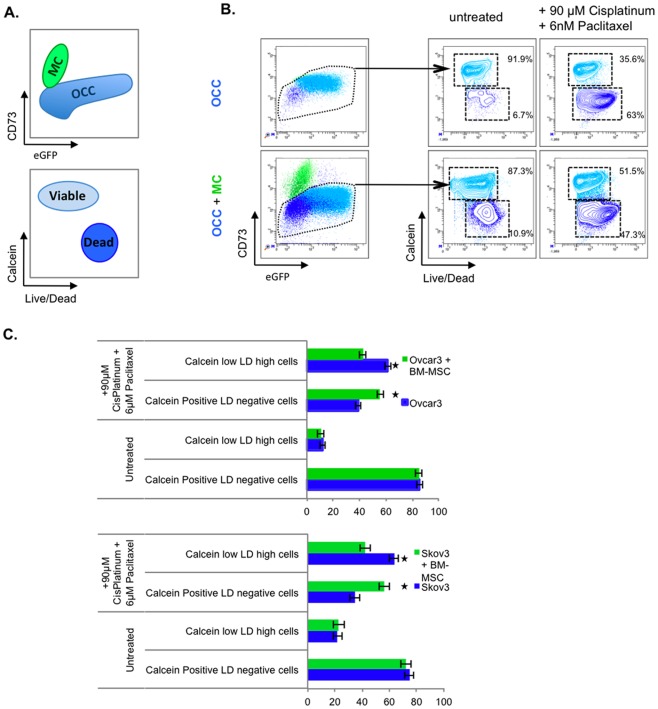
MC protects OCC from chemotherapy induced cell death. **A.** Explanative scheme of FACS analysis. **B**. OCC or OCC co-cultivated with MSC were treated for 48 hours with 90 µM of Cisplatin and 6 nm of Paclitaxel. Cell types were discriminated by FACS using GFP as a cancer cell marker, and CD73 as an MC marker. Cell death analysis was carried out using the dual Calcein/Live Dead staining. **C**. Quantitation of cell death analysis experiments. OCC display a resistance to conventional chemotherapy treatment when co-cultivated with MC compared to the control. SEM are represented, n = 3, | p<0.05 t-Student Test.

OCC were cultivated for 24 h in absence or presence of MC in a serum free, cytokine free media. The mono- or cocultures were then treated for 24 hours with 90 µM Cisplatinum and 6nm Paclitaxel. Prior FACS analysis, cells were stained with a viability dye (Calcein) and a cell death dye (LIVE/DEAD). OCC and MSC were discriminated on their differential expression of CD73 and eGFP, OCC were defined as eGFP+CD73- (figure 4.A, upper panel). Viable OCC after chemotherapy treatment was defined as Calcein High/Live Dead (LD) negative (figure 4.A, lower panel).

Co-culture of OCC with MC did not modify cell viability or mortality before chemotherapy (Figure 4.B and 4.C). Chemotherapeutic treatment resulted in 61.4% (NIH:OVCAR3) and 63.4% (SKOV3) of OCC death (Calcein low, LD high cells) (Figure 4.C). While OCC death was reduced after chemotherapy with 42.3% (NIH:OVCAR3) and 42% (SKOV3) of Calcein low, LD high cells in presence of MC (Figure 4.C).

In corroboration with our IPA results, we demonstrated that MC were not only able to enrich gene clusters that are involved in regulation of cell death, but were also promoting OCC resistance (1.45 and 1.50 fold decrease in cell death for NIH:OVCAR3 and SKOV3 respectively) to chemotherapy.

## Discussion

Our study demonstrated that MCs modify OCC transcriptome toward a metastatic profile. Upon contact with MC, OCC lines (NIH:OVCAR3 and SKOV3) enriched three biological function clusters: “Metastasis”, “Proliferation of cell lines”, “Cell death of tumor cell line”. We validated that these functions were modified *in vitro.* Our data indicated that MC contact with OCC increased adherence, migration and invasion to/through the ECM, proliferation, and resistance to chemotherapy. This is consistent with reports of MCs modifying ovarian cancer cells behavior [7], [8], [11], [12], [17].

The peritoneal cavity is lined by a continuous single layer of mesothelial cells [18]. Mesothelial cells are described in the literature as the first line of defense against all abdominally metastasizing tumors. Therefore, the development of peritoneal carcinosis in ovarian cancer involves several key steps: adhesion and clearance to/of the mesothelial layer, migration and invasion through the mesothelial and sub-mesothelial layers, and proliferation of the ovarian cancer cells [2], [19]. While mesothelial cells have been described to inhibit ovarian cancer cell adhesion and invasion [20], carcinoma-associated fibroblasts (CAF) display the opposite effects [12], [20]. In our study, we demonstrated that mesenchymal cells (MC) increased ovarian cancer cells adhesion and invasion. This is in line with Kenny and al data where they described mesenchymal cells-derived fibroblasts promote ovarian cancer cell migration and invasion in a MMP2 dependant manner [21]. Recently, they also showed the role of adipocytes in ovarian cancer progression [22]. Noteworthy, MC are described to differentiate into CAF and adipocytes when they are exposed to ovarian cancer cell supernatant [11], [17]. Taken together these data demonstrate that the mesenchymal lineage (MC, CAF, adipocytes) constitutes a permissive niche to ovarian cancer cells, and could explain the peritoneal tropism of ovarian tumor metastatic outgrowth.

In this study we demonstrated that MC sustain ovarian cancer cell proliferation in a serum free, cytokine free context. These data obtained in vitro, are in corroboration with several in vivo report. For instance, Pasquet and al. demonstrated that mesenchymal ascites derived stromal cells promote ovarian tumor growth in nude mice [9]. This ability of mesenchymal ascites derived stromal cells to promote ovarian tumor growth was explained by the induction of ovarian cancer cell proliferation, and by an increase of angiogenesis [9]. More recently, Mac Lean and al. showed that MC could also increase ovarian tumor heterogeneity. Altered secretion of BMP2 by MCs is able to increase the number of ovarian cancer stem cells and consequently stimulate ovarian tumor growth in an immuno-compromised mouse [12]. Another component of mesenchymal lineage, the adipocytes, was also reported to increase in vitro and in vivo tumor growth by providing energy to the cancer cells through the production of fatty acids [22]. The role of the fatty acid on the ovarian cancer cell biology is recently emerging. A recent work showed that chemoresistance were induced by MC through the release of platinium-induced fatty acids [23]. These fatty acid demonstrated the ability, in a context of xenograft, to protect OCC from numerous chemotherapeutic agents [23]. Since the main clinical issue regarding the ovarian disease is the relapse emerging from a platinum resistant minimal disease, it is interesting to note that MC and other mesenchymal components are able to drive both proliferation and resistance to therapy by providing a permissive “metabolic” niche.

Therefore MC and MC-derived cells determine both ovarian cancer biology and ovarian cancer response to therapy. Understanding gene expression changes that occur in OCC upon their interaction with their MC niche could help to define new therapeutic approaches. Our aim in this study was to describe a relationship between changes that occur in ovarian cancer cell line at the transcriptomic level and differences in cell behavior we observed with the coculture assay we performed. One limitation of this study is that compared transcriptomic analysis of NIH:OVCAR3 and SKOV3 in contact with MC failed to define a shared global change at the gene expression level. Recently the cancer genome atlas published their work on ovarian serous adenocarcinomas [24]. They were able to define four new subsets (differentiated, immunoreactive, mesenchymal and proliferative) of ovarian high-grade serous adenocarcinomas based on a gene expression study of 489 patients [24]. One explanation for the lack of overlap in gene expression changes between NIH:OVCAR3 and SKOV3 could be that each cell line belongs to different subset of ovarian high-grade serous adenocarcinomas. Indeed OVCAR3 and SKOV3 could represent different cell types based on the principal component analysis (Figure 1.B). Despite the apparent discrepancy, we were able to define three identical biological-function gene clusters enriched upon MC contact that corroborated our in vitro data. Surprisingly, the mesenchymal niche seems to be permissive to two different subsets of ovarian serous adenocarcinomas adhesion, migration, invasion, proliferation and chemoresistance. Further studies on the effect of the microenvironment on each different subset of ovarian serous adenocarcinomas (differentiated, immunoreactive, mesenchymal and proliferative) are therefore required to refine the genes involved and define innovative therapeutic approaches.

## Supporting Information

Table S1 Gene expression analysis. Fold changes represents the gene expression changes in NIH:OVCAR3 after contact with MC compared to the control.
(DOC)

Table S2 Gene expression analysis. Fold changes represents the gene expression changes in SKOV3 after contact with MC compared to the control.
(DOC)
