# Supplementary material for: Mesenchymal Cell Interaction with Ovarian Cancer Cells Triggers Pro-Metastatic Properties
Source: PLoS One. 2012 May 30;7(5):e38340. doi: 10.1371/journal.pone.0038340 (PMC3364218; doi:10.1371/journal.pone.0038340)
Supplement: Table S1 — Gene expression analysis. Fold changes represents the gene expression changes in NIH:OVCAR3 after contact with MC compared to the control. (DOC) [file pone.0038340.s001.doc]

| Symbol | Entrez Gene Name | Affymetrix | Fold Change | | Entrez Gene ID for Human | | Entrez Gene ID for Mouse | Entrez Gene ID for Rat |
| --- | --- | --- | --- | --- | --- | --- | --- | --- |
| COL1A2 | collagen, type I, alpha 2 | 202404_s_at | -50.165 |  | | 1278 | 12843 | 84352 |
| SPARC | secreted protein, acidic, cysteine-rich (osteonectin) | 200665_s_at | -11.110 |  | | 6678 | 20692 | 24791 |
| FN1 | fibronectin 1 | 211719_x_at | -10.982 |  | | 2335 | 14268 | 25661 |
| COL6A3 | collagen, type VI, alpha 3 | 201438_at | -9.575 |  | | 1293 | 12835 | 367313 |
| COL1A1 | collagen, type I, alpha 1 | 1556499_s_at | -9.175 |  | | 1277 | 12842 | 29393 |
| GREM1 | gremlin 1 | 218469_at | -8.078 |  | | 26585 | 23892 | 50566 |
| POSTN | periostin, osteoblast specific factor | 210809_s_at | -7.966 |  | | 10631 | 50706 | 361945 |
| HSPA8 | heat shock 70kDa protein 8 | 210338_s_at | -7.289 |  | | 3312 | 15481 | 24468 |
| COL3A1 | collagen, type III, alpha 1 | 201852_x_at | -6.646 |  | | 1281 | 12825 | 84032 |
| HSPA1A/HSPA1B | heat shock 70kDa protein 1A | 202581_at | -5.105 |  | | 3303|3304 | 193740|15511 | 294254|24472 |
| RPS6KA2 | ribosomal protein S6 kinase, 90kDa, polypeptide 2 | 212912_at | 5.107 |  | | 6196 | 20112 | 117269 |
| FYN | FYN oncogene related to SRC, FGR, YES | 210105_s_at | 5.237 |  | | 2534 | 14360 | 25150 |
| GOT1 | glutamic-oxaloacetic transaminase 1, soluble (aspartate aminotransferase 1) | 208813_at | 5.261 |  | | 2805 | 14718 | 24401 |
| CEBPB | CCAAT/enhancer binding protein (C/EBP), beta | 212501_at | 5.386 |  | | 1051 | 12608 | 24253 |
| CCND2 | cyclin D2 | 200953_s_at | 5.423 |  | | 894 | 12444 | 64033 |
| BCL6 | B-cell CLL/lymphoma 6 | 203140_at | 5.544 |  | | 604 | 12053 | 303836 |
| SEL1L3 | sel-1 suppressor of lin-12-like 3 (C. elegans) | 212314_at | 5.560 |  | | 23231 | 231238 | 360945 |
| LARP6 | La ribonucleoprotein domain family, member 6 | 218651_s_at | 5.563 |  | | 55323 | 67557 | 315731 |
| DDIT4 | DNA-damage-inducible transcript 4 | 202887_s_at | 6.046 |  | | 54541 | 74747 | 140942 |
| CHAC1 | ChaC, cation transport regulator homolog 1 (E. coli) | 219270_at | 7.375 |  | | 79094 | 69065 | 362196 |
| C9orf150 | chromosome 9 open reading frame 150 | 227443_at | 7.413 |  | | 286343 | 52829 | 362535 |
| KLF9 | Kruppel-like factor 9 | 203543_s_at | 7.605 |  | | 687 | 16601 | 117560 |
| KLHL24 | kelch-like 24 (Drosophila) | 226158_at | 8.177 |  | | 54800 | 75785 | 303803 |
| GADD45A | growth arrest and DNA-damage-inducible, alpha | 203725_at | 8.742 |  | | 1647 | 13197 | 25112 |
| TSC22D3 | TSC22 domain family, member 3 | 208763_s_at | 9.287 |  | | 1831 | 14605 |  |
| PCK2 | phosphoenolpyruvate carboxykinase 2 (mitochondrial) | 202847_at | 9.350 |  | | 5106 | 74551 | 361042 |
| DDIT3 | DNA-damage-inducible transcript 3 | 209383_at | 9.775 |  | | 1649 | 13198 | 29467 |
| TXNIP | thioredoxin interacting protein | 201010_s_at | 9.983 |  | | 10628 | 56338 | 117514 |
| C5orf41 | chromosome 5 open reading frame 41 | 225956_at | 12.141 |  | | 153222 | 77128 | 303016 |
| ATF3 | activating transcription factor 3 | 202672_s_at | 12.425 |  | | 467 | 11910 | 25389 |
| CTH | cystathionase (cystathionine gamma-lyase) | 206085_s_at | 14.916 |  | | 1491 | 107869 | 24962 |
| TRIB3 | tribbles homolog 3 (Drosophila) | 218145_at | 14.920 |  | | 57761 | 228775 | 246273 |
| SLC7A11 | solute carrier family 7 (anionic amino acid transporter light chain, xc- system), member 11 | 217678_at | 15.653 |  | | 23657 | 26570 | 310392 |
| ASNS | asparagine synthetase (glutamine-hydrolyzing) | 205047_s_at | 23.756 |  | | 440 | 27053 | 25612 |
| GDF15 | growth differentiation factor 15 | 221577_x_at | 27.920 |  | | 9518 | 23886 | 29455 |
| INHBE | inhibin, beta E | 210587_at | 38.306 |  | | 83729 | 16326 | 83711 |
