# Supplementary material for: Mesenchymal Cell Interaction with Ovarian Cancer Cells Triggers Pro-Metastatic Properties
Source: PLoS One. 2012 May 30;7(5):e38340. doi: 10.1371/journal.pone.0038340 (PMC3364218; doi:10.1371/journal.pone.0038340)
Supplement: Table S2 — Gene expression analysis. Fold changes represents the gene expression changes in SKOV3 after contact with MC compared to the control. (DOC) [file pone.0038340.s002.doc]

| Symbol | Entrez Gene Name | Affymetrix | Fold Change | | Entrez Gene ID for Human | | Entrez Gene ID for Mouse | Entrez Gene ID for Rat | |
| --- | --- | --- | --- | --- | --- | --- | --- | --- | --- |
| CLIC3 | chloride intracellular channel 3 | 219529_at | -7.46 |  | | 9022 | 69454 | 296566 |  |
| ALDOC | aldolase C, fructose-bisphosphate | 202022_at | -5.613 |  | | 230 | 11676 | 24191 |  |
| UCP2 | uncoupling protein 2 (mitochondrial, proton carrier) | 208998_at | -5.162 |  | | 7351 | 22228 | 54315 |  |
| IL33 | interleukin 33 | 209821_at | 5.069 |  | | 90865 | 77125 | 361749 |  |
| MMP3 | matrix metallopeptidase 3 (stromelysin 1, progelatinase) | 205828_at | 5.878 |  | | 4314 | 17392 | 171045 |  |
| SPARC | secreted protein, acidic, cysteine-rich (osteonectin) | 212667_at | 6.46 |  | | 6678 | 20692 | 24791 |  |
| UQCRH | ubiquinol-cytochrome c reductase hinge protein | 202233_s_at | 6.711 |  | | 7388 | 100042918 | |  |
| CLDN11 | claudin 11 | 228335_at | 6.752 |  | | 5010 | 18417 | 84588 |  |
| CDH11 | cadherin 11, type 2, OB-cadherin (osteoblast) | 207173_x_at | 7.012 |  | | 1009 | 12552 | 84407 |  |
| PAPPA | pregnancy-associated plasma protein A, pappalysin 1 | 228128_x_at | 7.33 |  | | 5069 | 18491 | 313262 |  |
| C11orf96 | chromosome 11 open reading frame 96 | 227099_s_at | 7.468 |  | | 387763 | 620695 | 499839 |  |
| SFRP4 | secreted frizzled-related protein 4 | 204051_s_at | 7.872 |  | | 6424 | 20379 | 89803 |  |
| CCL2 | chemokine (C-C motif) ligand 2 | 216598_s_at | 8.275 |  | | 6347 | 20293 | 287562 |  |
| GREM1 | gremlin 1 | 218469_at | 9.799 |  | | 26585 | 23892 | 50566 |  |
| KIAA1199 | KIAA1199 | 212942_s_at | 9.958 |  | | 57214 | 80982 | 308797 |  |
| COL6A3 | collagen, type VI, alpha 3 | 201438_at | 10.121 |  | | 1293 | 12835 | 367313 |  |
| CXCL6 | chemokine (C-X-C motif) ligand 6 (granulocyte chemotactic protein 2) | 206336_at | 15.511 |  | | 6372 | 20311 | 60665 |  |
| MGP | matrix Gla protein | 202291_s_at | 16.068 |  | | 4256 | 17313 | 25333 |  |
| COL3A1 | collagen, type III, alpha 1 | 215076_s_at | 24.528 |  | | 1281 | 12825 | 84032 |  |
| COL1A1 | collagen, type I, alpha 1 | 1556499_s_at | 31.15 |  | | 1277 | 12842 | 29393 |  |
| COL1A2 | collagen, type I, alpha 2 | 202404_s_at | 50.41 |  | | 1278 | 12843 | 84352 |  |
